# Supplementary material for: Which character strengths may build organizational well-being? Insights from an international sample of workers
Source: PLoS One. 2024 Oct 30;19(10):e0312934. doi: 10.1371/journal.pone.0312934 (PMC11524506; doi:10.1371/journal.pone.0312934)
Supplement: S1 Table — (DOCX) [file pone.0312934.s001.docx]

**Supplementary materials accompanying the manuscript**

**Which character strengths may build organizational well-being? Insights from an international sample of workers**

**Table S1**

*Overview of the presented well-being theories*

| **Model** | **Dimensions** | **Features** |
| --- | --- | --- |
| **Subjective Well-being (SWB)** | Cognitive | Satisfaction with life |
|  | Affective | Positive and negative affect |
| **Psychological Well-being (PWB)** | Self-acceptance | Positive attitude towards oneself and one’s past |
|  | Environmental mastery | Competence |
|  | Positive relations | Close and trustful interpersonal relationships |
|  | Autonomy | Self-regulation |
|  | Purpose in life | Meaning in life |
|  | Personal growth | Improvement over time |
| **PERMA** | Positive emotion | Tendency to experience joy and contentment |
|  | Engagement | Feel involved with activities and experience flow |
|  | Relationships | Connect with others, feeling loved and appreciated |
|  | Meaning | Sense of purpose and direction in life |
|  | Accomplishment | Mastery, achievement |
| **PERMA + 4** | Physical Health | Biological health |
|  | Mindset | Future orientation, growth mindset, perseverance |
|  | Environment | Access to external resources (e.g., light, nature) |
|  | Economic Security | Perception of financial steadiness |
