# Supplementary material for: Which character strengths may build organizational well-being? Insights from an international sample of workers
Source: PLoS One. 2024 Oct 30;19(10):e0312934. doi: 10.1371/journal.pone.0312934 (PMC11524506; doi:10.1371/journal.pone.0312934)
Supplement: S2 Table — Adapted from Peterson and Seligman (2004, pp. 29–30). (DOCX) [file pone.0312934.s002.docx]

**Supplementary materials accompanying the manuscript**

**Which character strengths may build organizational well-being? Insights from an international sample of workers**

**Table S2.**

*The VIA classification. Adapted from Peterson and Seligman (2004, pp. 29-30)*

| **Core virtue** | **Corresponding character strengths** |
| --- | --- |
| **Wisdom:**  **Acquire and use knowledge** | Creativity [originality, ingenuity]  Curiosity [interest, novelty-seeking, openness to experience]  Judgement [open-mindedness, critical thinking]  Love of learning [systematically adding knowledge]  Perspective [wisdom] |
| **Courage: Pursue goals despite adversity** | Bravery [valor]  Perseverance [persistence, industriousness]  Honesty [authenticity, integrity]  Zest [vitality, enthusiasm, vigor, energy] |
| **Humanity: Care for other people** | Love [closeness, intimacy]  Kindness [generosity, nurturance, care, compassion]  Social intelligence [emotional intelligence, personal intelligence] |
| **Justice: Care for the community** | Teamwork [citizenship, social responsibility, loyalty]  Fairness  Leadership |
| **Temperance: Resist excesses** | Forgiveness [mercy]  Humility [modesty]  Prudence [cautiousness]  Self-regulation [self-control] |
| **Transcendence: Connect with a purpose** | Appreciation of beauty and excellence [awe, wonder]  Gratitude [thankfulness]  Hope [optimism, future-mindedness, future orientation]  Humor [playfulness]  Spirituality [religiousness, faith, purpose] |
