# Supplementary material for: Which character strengths may build organizational well-being? Insights from an international sample of workers
Source: PLoS One. 2024 Oct 30;19(10):e0312934. doi: 10.1371/journal.pone.0312934 (PMC11524506; doi:10.1371/journal.pone.0312934)
Supplement: S3 Table — Note. ß = standardized beta coefficient; * p < .05, ** p < .01, *** p < .001. (DOCX) [file pone.0312934.s003.docx]

**Supplementary materials accompanying the manuscript**

**Which character strengths may build organizational well-being? Insights from an international sample of workers**

**Table S3.**

*Linear multiple regression with PERMA+4 and character strengths as predictors, and life satisfaction as outcome*

| Predictor | *β* |
| --- | --- |
| Appreciation of beauty | −.06*** |
| Bravery | −.06*** |
| Creativity | −.07*** |
| Curiosity | .09*** |
| Fairness | .02 |
| Forgiveness | −.04** |
| Gratitude | .28*** |
| Honesty | .04*** |
| Hope | .09*** |
| Humility | −.03** |
| Humor | −.02 |
| Judgment | −.02 |
| Kindness | −.06** |
| Leadership | .06*** |
| Love | .08*** |
| Love of learning | −.08*** |
| Perseverance | −.02 |
| Perspective | .01 |
| Prudence | .04* |
| Self-regulation | .02 |
| Social intelligence | −.03* |
| Spirituality | −.10*** |
| Teamwork | −.05*** |
| Zest | .03* |
| PERMA+4 | .49*** |
| R^2^ | .49 |

*Note. ß* = standardized beta coefficient; * *p* < .05, ** *p* < .01, *** *p* < .001
