# Supplementary material for: Which character strengths may build organizational well-being? Insights from an international sample of workers
Source: PLoS One. 2024 Oct 30;19(10):e0312934. doi: 10.1371/journal.pone.0312934 (PMC11524506; doi:10.1371/journal.pone.0312934)
Supplement: Attachment — Submitted filename: Response to Reviewers.docx [file pone.0312934.s004.docx]

**Revision Notes**

**Reviewer 1**

The present study analyses character strength (24 strength and a general factor) in relation to well-being (PERMA+4) and satisfaction in a large sample of 5,487 employees. The manuscript quotes the relevant articles and develops toward the 4 research questions posed. Next to zero order correlations also a mediation analysis was performed to see whether PERMA+4 mediates the relation between character strength and life satisfaction.

**REPLY**: Thank you for taking the time to review our manuscript and for making suggestions on how to improve it further.

Regarding the zero order correlations it is not surprising that strengths correlate more highly with the PERMA total score than life satisfaction does. This could be due to both higher reliability (due to higher number of items) and broader content (which reduces the number of low correlations. So the statement that “we found that 21 of the 24 character strengths were more strongly correlated with PERMA+4 than with life satisfaction” needs to be reevaluated. Likewise, the authors should consider using strength and PERMA in a normal regression analysis to acknowledge and that you have cross-sectional data. You say that you need a longitudinal design but as long as you don’t have one it is safer to avoid mediation analysis. Maybe predictors (and an interaction) would also yield interesting results.

**REPLY**: Thank you for pointing these important considerations out. In our sample, both SWLS and PERMA showed similarly high reliability (.88 and .91, respectively); nevertheless, we agree that the current statement may be too definitive, and decided to modify it as follows (see the modifications in red): “We found that 21 of the 24 character strengths were significantly (yet only slightly) more strongly correlated with PERMA+4 than with life satisfaction” (see line 35). Regarding the mediation vs. regression analysis, we opted for a SEM approach to be able to also specify the measurement model for the variables and add mediation effects between each character strength and PERMA+4, while also disentangling the variance explained by specific strengths and the general factor of character (Model 2). We nevertheless decided to include a multiple regression analysis in the supplementary materials (Table S3) with the 24 strengths and the PERMA+4 as predictors and SWLS as outcome. The results are very similar to the ones obtained in the SEM model, as now specified in the main text as well: “We also fitted a multiple linear regression model with the 24 character strengths and PERMA+4 as independent variables and SWLS as dependent variable and obtained similar results, with hope, love, and zest also showing small positive effects on life satisfaction (see Table S3).” (see lines 319-322)

A few minor questions:
You talk about SWB (which included life satisfaction, and affect balance) but you only measure satisfaction with life? Why not including positive and negative affect as well? Or avoid SWB altogether?

**REPLY**: Thank you for pointing this out. We only focused on the cognitive component of subjective well-being (without considering positive and negative affect) as it’s the main indicator used in the literature and due to the limited number of additional items that can be administered through the VIA website, to avoid excessive fatigue and careless responding. We recognize that the reference to SWB only may be misleading, and we therefore decided to avoid it whenever possible (see changes throughout the manuscript). We also now list the lack of the affective component of subjective well-being as a limitation of our present work: “Future work should also consider domain-specific forms of satisfaction, such as job satisfaction, and positive and negative affect as a relevant outcome that may be associated with both character strengths and PERMA+4.” (see lines 474-476)

You use the term “building block” often, even “building blocks of the building blocks”. Can you elaborate what building block implies or means. As far as I know it is not a technical term in psychology, nor a widely used expression with a clear meaning.

**REPLY**: Thank you for giving us the opportunity to clarify this further. We used the expression “building blocks” to refer to the paper by Seligman (2018) “PERMA and the building blocks of well-being”. In the paper, the author uses this term to indicate that PERMA dimensions represent the constitutive elements of well-being, rather than a distinct new type of well-being. In the present paper, we additionally hypothesize that character strengths may represent the constitutive elements (building blocks) of PERMA, or, said differently, more distal predictors of life satisfaction. We now made this reference more explicit in the paper adding the following clarifications: “In his response, the author clarified that PERMA does not in fact constitute a new type of well-being, but rather the “building blocks” (as he calls them) that lead to SWB” (line 87); “We speculate that the PERMA+4 dimensions (the building blocks of work-related well-being in Seligman’s view)” (line 126); “In this sense, character strengths could be considered as the “building blocks of the building blocks” of well-being, or, put differently, as more distal predictors of life satisfaction compared to PERMA+4.” (line 137); “The present study aims to make an initial contribution to the recent debate on well-being and its constitutive elements, or “building blocks”” (line 170).

You write “It is historically distinct from psychological well-being (PWB), which instead represents the eudaimonic type of well-being, i.e., a focus on growth and optimal psychological functioning, with a greater attention to the interpersonal dimension (4).” SWB was there before PWB; therefore it is misleading or at least imprecise to say it Is “historically distinct”.

**REPLY:** Thank you for noticing this imprecision. We rephrased the sentence as follows to avoid misleading the reader: “It is commonly considered in the literature as distinct from psychological well-being (PWB) (…)” (see lines 67-68).

You write “In both models, life satisfaction (modelled as a latent variable) was the dependent variable, and Positive Functioning at Work (also modelled as a unidimensional latent variable) was the mediator.”

“Table 5. Results of the first path model (M1), with character strengths as predictors, Positive
Functioning at Work as mediator, and life satisfaction as dependent variable“ Why did you nt consider work satisfaction?

**REPLY:** Thank you for bringing this up. Since our work is, to the best of our knowledge, the first examination of character strengths and PERMA+4 (or Positive Functioning at Work, as the authors also call it), we decided to focus on general life satisfaction. Future work should definitely consider domain-specific satisfaction, such as work satisfaction, as an interesting outcome. We added this future direction in the Discussion section by saying the following: “Future work should also consider domain-specific forms of satisfaction, such as job satisfaction, and positive and negative affect as a relevant outcome that may be associated with both character strengths and PERMA+4.” (see lines 474-476).

**Reviewer 2**

Reviewer #2: Dear Authors,
The article presents a relevant theme, given that the construct of well-being has been the object of studies and research aimed at understanding the phenomenon and, consequently, subsidizing the construction of public policies, as well as organizational policies and guidelines, in order to favours people's happiness in various dimensions of their lives. Research on the PERMA model is still scarce, which gives the study an innovative character.

**REPLY**: Thank you for taking the time to review our work and for providing us with suggestions on how to improve it further.

The article presents a number of positive points, namely:
-The title fully corresponds to the study presented, in addition to presenting an adequate number of words.
-The abstract presents the required elements, such as objectives, methodology, scope, sample, main results and conclusions.
-The introduction denotes conceptual elements necessary for the formulation of the problem, as well as evidencing the objectives of the study. Elements that show the relevance of the subject addressed are also presented. This paper presents a review of the current literature.
- The large international sample.
-The methodology is consistent with the study's proposal. Statistical analyses are appropriate to the proposed objectives.

**REPLY**: Thank you for appreciating the merits of our manuscript.

However, it is recommended that authors make some changes to the article for publication.
The suggestions for change/improvement are as follows:
- In the introduction, Table 1(Overview of the presented well-being theories). and Table 2 (The VIA classification. Adapted from Peterson 156 and Seligman ((2), pp. 29-30) should be removed.

**REPLY**: Thank you for the suggestion. We decided to move the two tables to the supplementary materials, where they could be still of help for readers who are not familiar with the topic.

- In the introduction, between lines 135 to 154, authors must present the information in a narrative form and not by topics.

**REPLY**: Thank you for your comment. We now present the information narratively: “Looking more closely at the criteria proposed by Seligman (14) for assessing potential elements of well-being, we could argue that character strengths meet most of them. First of all, there is accumulating evidence that character strengths are significantly associated with both life satisfaction (13,33) and PERMA (12,13), which would fulfill the criterion “contribute to well-being”. Then, for the criterion “to be pursued for their own sake (rather than as means to an end)”, strengths are, by definition, intrinsically morally valued and do not depend on potential positive outcomes to be classified as such (2), as also shown empirically (34). Although the classification has been subject to criticism and revision (35), it was also developed after intensive historical, cross-cultural, and philosophical review, making it rather comprehensive, which would speak in favor of the taxonomy being “exclusive and exhaustive”. As briefly mentioned, there are a variety of character strengths based interventions (27,35), and there is strong evidence for their efficacy on life satisfaction (26), with some encouraging evidence of their ability to increase strengths trait levels (37). This evidence would support that character strengths can indeed be translated into specific interventions to build each other as well as well-being. Although 24 traits are quite a large number of variables to be considered, strengths can be considered as a unique factor (17,22), as recently reviewed by McGrath (18, and see next paragraph for a more detailed description), making the list more parsimonious. Lastly, although strengths are highly correlated with each other, they can be assessed separately using the VIA-IS questionnaires (2) and have shown differential correlations with well-being indicators. As such, they can be defined and measured independently.” (see lines 138-156).

- In the introduction, it would be relevant for the authors to present the concept/perspective of organizational well-being inherent to their research;

**REPLY**: Thank you the important suggestion. We now better integrated the concept of organizational well-being in the introduction: “Organizational well-being therefore here refers to both the eudaimonic (PERMA+4 dimensions) and hedonic (life satisfaction) components of well-being experienced by workers, in line with established frameworks (32). These different kinds of well-being represent “what” constitutes organizational well-being, while strengths have to do with “how” to enhance well-being in the workplace (32).” (see lines 131-135).

- The ethical procedures of the investigation must be better explained.

**REPLY**: Following this comment, we now expanded on the ethical aspects of our study further: “No IRB approval was required according to the University of Padova Ethics Committee, since the data was collected through an independent, international body (i.e., the VIA Institute). Although no informed consent was formally required, participants were informed of the VIA Institute’s privacy policy (<https://www.viacharacter.org/privacy-policy>, which they could access at any time by clicking at the bottom of each page) and could opt out of the survey at any moment and skip any questions they did not feel comfortable answering. For instance, they were informed that if they opted in to participate in the study, the VIA Institute could share the de-identified results (i.e., without the participant’s name or email address) with the researchers.” (see lines 201-208).

- In the results there is some repetition between the data presented in Table 4. (Correlations of character strengths, virtues, and character with PERMA+4) and the text between Line 281 and Line 301. Thus, it is suggested to synthesize the text between Line 281 to Line 301 and only the most relevant correlations are mentioned in the text.

**REPLY**: Following this suggestion, we now streamlined the text and only highlighted the main correlations: “For positive emotions, three character strengths showed correlations above .30 (zest: *r* = .48, hope: *r* = .45; gratitude: *r* = .39). For engagement, the correlations were mostly very modest, with only love of learning (*r* = .29) and curiosity (*r* = .23) showing correlations above .20. For relationships, two correlations were above .30 (teamwork: *r* = .33, hope: *r* = .32). For meaning, four correlations resulted stronger than .30 (hope: *r* = .36, gratitude: *r* = .34, zest: *r* = .32, and spirituality: *r* = .31). For accomplishment, the correlations were generally stronger, with eight correlations exceeding .30 (with the top three correlated strengths being hope: *r* = .47, perseverance: *r* = .45, and self-regulation: *r* = .40). With respect to the additional four PERMA dimensions, the correlations were mostly small. For physical health, four correlations exceeded .30 (zest: *r* = .38, hope: *r* = .38, self-regulation: *r* = .35, and gratitude: *r* = .30). In terms of mindset, three strengths showed a correlation above .30 (hope: *r* = .39, zest: *r* = .35, and gratitude: *r* = .31). For environment, only four correlations were between .20 and .30 (zest: *r* = .27, hope: *r* = .27, gratitude: *r* = .25, and perseverance: *r* = .20). Finally, the correlations for economic security were very low, except for a small correlation with self-regulation (*r* = .21).” (see lines 285-298).

- Authors should present the limitations inherent to their research.

**REPLY**: Following this and the other reviewer’s comments, we now spelled out the study’s limitations more explicitly in the Discussion section: “The present study also has some limitations. First, the cross-sectional nature of the study does not allow causality to be inferred. Future studies should examine these relationships longitudinally, to assess whether character strengths and PERMA+4 actually predict life satisfaction over time, not just cross-sectionally. Second, we only focused on domain-general satisfaction, and only on the cognitive component of subjective well-being. Future work should also consider domain-specific forms of satisfaction, such as job satisfaction, and positive and negative affect as a relevant outcome that may be associated with both character strengths and PERMA+4.” (see lines 470-476).

- In the discussion, it is important that the authors present the implications of their research for the development of policies and practices that promote organizational well-being.

**REPLY**: Thank you for bringing this up. We now detailed the practical implications of our research in the Discussion section: “These findings have practical implications, at the organizational and individual levels. They suggest that promoting the awareness and development of PERMA+4 elements in organizational contexts may have effects on the employees life satisfaction, and that helping them be aware, explore, and apply (36) their character qualities through formal (character strengths-based interventions) and informal (by highlighting them as core organizational values) practices may further strengthen their well-being.” (see lines 464-469).

In general, I would like to highlight the interest of the work submitted for publication and I congratulate the authors on their work. However, I believe that these changes are necessary to improve its final quality and make it attractive to readers.

**REPLY**: Thank you for your helpful comments, we integrated all of them in the manuscript.
